# Life, death, and autophagy in cancer: NF-κB turns up everywhere

**DOI:** 10.1038/s41419-020-2399-y

**Published:** 2020-03-30

**Authors:** Daniela Verzella, Alessandra Pescatore, Daria Capece, Davide Vecchiotti, Matilde Valeria Ursini, Guido Franzoso, Edoardo Alesse, Francesca Zazzeroni

**Affiliations:** 1 Department of Biotechnological and Applied Clinical Sciences (DISCAB), University of L’Aquila, 67100 L’Aquila, Italy; 2 Institute of Genetics and Biophysics “Adriano Buzzati-Traverso”, IGB-CNR, Naples, 80131 Italy; 3 Centre for Molecular Immunology and Inflammation, Department of Immunology and Inflammation, Imperial College London, London, W12 0NN UK

**Keywords:** Cancer, Cell death

## Abstract

Escaping programmed cell death is a hallmark of cancer. NF-κB transcription factors are key regulator of cell survival and aberrant NF-κB signaling has been involved in the pathogenesis of most human malignancies. Although NF-κB is best known for its antiapoptotic role, other processes regulating the life/death balance, such as autophagy and necroptosis, seem to network with NF-κB. This review discusses how the reciprocal regulation of NF-κB, autophagy and programmed cell death affect cancer development and progression.

## Facts

- NF-κB transcription factors are key regulator of cell survival and aberrant NF-κB signaling has been involved in the pathogenesis of most human malignancies.
- NF-κB is well known for providing cancer cells with a survival advantage by upregulating antiapoptotic genes.
- There is a reciprocal crosstalk between NF-κB and autophagy in cancer, where it can either promote or repress tumorigenesis, depending on the stimulus and the context.
- NF-κB proinflammatory signaling downstream of RIPK1/RIPK3 activation is required for the immunogenicity of necroptotic cells in the tumor-microenvironment (TME).

## Open questions

- Targeting autophagy and NF-κB as a strategy for improving anticancer therapy and bypass drug-resistance.
- The controversial role of endogenous necroptotic signaling within the tumor cell and its crosstalk with NF-κB pathway.
- The reciprocal interplay between autophagy and programmed cell death (PCD) in cancer and how these processes intersect with NF-κB signaling.

## Introduction—historical background

The choice of whether to live or die for the cell may depend on the activation of NF-κB transcription factors. The antiapoptotic function of NF-κB has long been established, since Beg and Baltimore described that *RelA*-deficient mice succumbed during embryogenesis due to massive apoptosis of hepatocytes^1^. Beyond its role in liver, the NF-κB pro-survival function is also crucial for the physiology of the immune system, where the constitutive activation of NF-κB pathway is needed for differentiation and maintenance of B lymphocytes, the development of thymocytes, as well as for immune response to antigens by mature B and T lymphocytes^2,3^.

The suppression of apoptosis by NF-κB could be viewed as a transcriptional event. NF-κB exerts its pro-survival activity by inducing the transcription of several antiapoptotic genes, and this transcriptional program seems to be specifically tailored depending on the tissues involved and the biological contexts. The NF-κB-mediated resistance to tumor necrosis factor-alpha (TNF-α)-induced apoptosis has been associated to the upregulation of several pro-survival genes, including cellular inhibitor of apoptosis 1 (cIAP1) and 2 (cIAP2), X chromosome-linked inhibitor of apoptosis (XIAP), TNF-R associated factor 1 (TRAF1) and 2 (TRAF2), cellular FLICE inhibitor protein (c-FLIP) and several Bcl-2 family members (i.e*.*, Bcl-X_L_ and A1/Bfl-1), mainly implicated in the inhibition of the apoptotic signaling^4–6^. However, these factors are not sufficient to account for the complete NF-κB-dependent blockade of apoptotic pathway. Over the past decades, NF-κB’s role in dampening JNK activation, both downstream of TNFR1 and other death stimuli, has been extensively investigated^7–10^. Indeed, the blockage of NF-κB pathway, by either ablation of RelA or IKKβ or overexpression of the super repressor IκBαM, caused the sustained and prolonged activation of JNK in response to TNF-α or concavalin A, and the persistence of this induction was responsible for cell death^7,8,10^. NF-κB represses sustained JNK activation both directly, by upregulating the expression of critical JNK inhibitors, and indirectly, via opposing oxidative stress. The NF-κB target gene encoding for Growth arrest and DNA-damage-inducible (Gadd45)β protein is one of the best-known suppressor of JNK pathway. Gadd45β antagonizes TNF-α-induced cytotoxicity by targeting the mitogen-activated protein kinase (MAPK) kinase (MKK)7, which in turn is responsible for JNK activation^7,11^. NF-κB also blocks JNK-dependent cell death by counteracting reactive oxygen species (ROS) accumulation, which in turn triggers JNK activation. Indeed, well established targets of NF-κB are several antioxidant factors, such as Ferritin heavy chain (FHC), manganese superoxide dismutase (MnSOD), Glutathione -S-transferase (GST) and metallothionein (MT)^12–16^.

Malignant cells often hijack key pathways and the same molecular networks that control normal development, and NF-κB pathway is no exception to the rule. Malignant cells are “addicted” to NF-κB signaling, which drives oncogenesis, disease recurrence and therapy resistance in both solid and haematological malignancies, where it induces transcriptional programmers sustaining all hallmarks of cancer^6,17–23^.

**Fig. 1:**
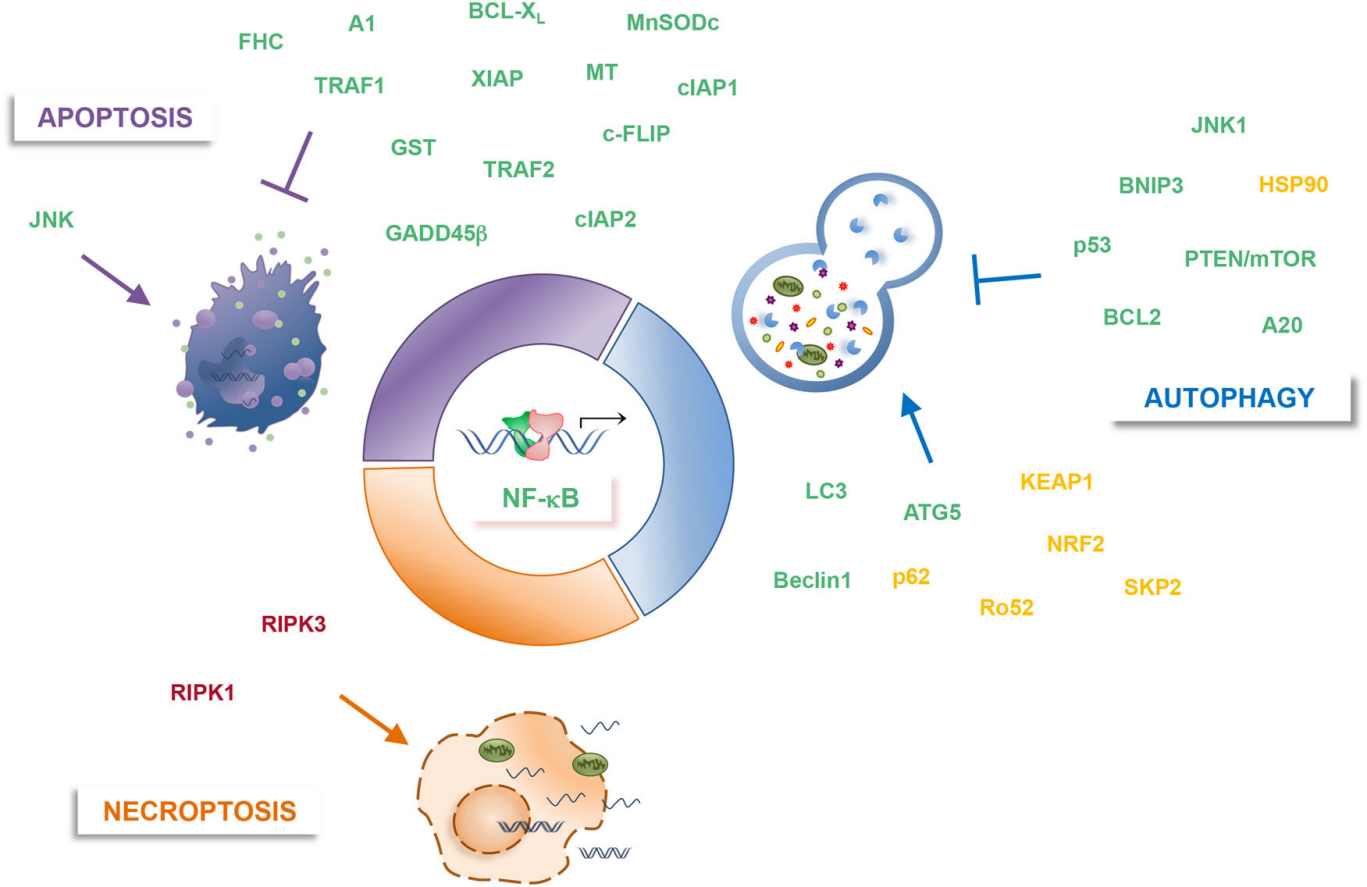
NF-κB involvement in autophagy and PCD. Shown are key positive (↑) or negative (⊥) modulators of autophagic, apoptotic and necroptotic processes, which are regulated by (green, red), or regulator of (yellow, red) NF-κB.

Multiple studies revealed that in the bulk of tumors in which NF-κB is constitutively active, it frequently provides cancer cells with a survival advantage by upregulating antiapoptotic genes^17,21^. Several evidence, both in vitro and in vivo, demonstrated that NF-κB antiapoptotic signaling is required for RAS-induced transformation^6,20,24,25^. In addition, seminal papers reported how the suppression of IKK/NF-κB signaling in mouse models of inflammation-driven colorectal (CRC) and hepatocellular carcinoma (HCC) resulted in apoptosis of preneoplastic cells and failure of tumor progression, thus establishing a key role for NF-κB as a linchpin between inflammation and cancer^26,27^. NF-κB pro-survival function is also critical in inflammation-driven tumor progression, as demonstrated in syngeneic colon and breast cancer xenograft models, where inflammation-associated NF-κB activation mediates tumor growth by conferring resistance to TRAIL death cytokine^28^. NF-κB constitutive activation is considered to be a signature also in several haematological malignancies, and in many of them, including activated B‐cell‐like (ABC)-diffuse large B‐cell lymphoma (DLBCL) and multiple myeloma (MM), it promotes cancer cell survival^17,21,29^. Recently, we furthered the understanding in MM, where we showed that oncogenic NF-κB signaling mediates cancer cell survival by upregulating *GADD45B*, which, in turn, suppresses apoptosis ensuing from JNK/MAPK-pathway activation by inhibiting MKK7 (Fig. 1)^30–32^.

While the role of NF-κB signaling in evading cancer cell apoptosis has been well-established, increasing evidence are pointing out for a broader role for NF-κB in balancing life and death. Autophagy and necroptosis play a key part in this scenario, and accordingly, NF-κB has been involved in their regulation. This review will focus on the role of this transcription factor in controlling autophagy and necroptosis in the context of cancer.

## Autophagy and NF-κB

Autophagy or “self-eating” is a physiological tightly conserved multistep process for recycling endogenous or exogenous cytoplasmatic materials like misfolded proteins, lipids, organelles (mitochondria, ribosomes), cellular components, peroxisomes and viruses or bacteria, that culminates with lysosomal degradation^33–35^. Three different types of autophagy co-exist in most cell types: microautophagy and endosomal microautophagy, chaperone-mediated autophagy (CMA) and macroautophagy^34^. The best characterized form of autophagy is macroautophagy, that relies on the formation of the autophagosome, a double-membraned vesicle able to take up the autophagic cargo, including protein aggregates and entire organelles. Depending on the specific autophagic substrate and initiating stimulus, macroautophagy can be defined selective (i.e*.*, mitophagy, lysophagy) or non-selective (intracellular materials)^34^. The selective recognition and recruitment of cargoes is driven by autophagic receptors, like sequestosome 1 (SQSTM1 or p62) autophagy-related proteins (ATG) and nuclear receptor binding factor 2 (NRBF2)^34^. The autophagic mechanism is highly regulated. Each phase involves specific regulatory molecules and complexes, such as mammalian target of rapamycin complex 1 (MTORC1), ubiquitin-like conjugation systems (i.e*.*, ULK1/Atg13/FIP200 and Beclin-1/Vps34/Atg14), microtubule-associated protein 1 light chain 3 (LC3), ATG7 and lysosomal-associated membrane proteins (i.e*.*, LAMP)^33,34,36^.

In addition to regulating cell survival, apoptosis and activation of inflammation, the NF-κB family of transcription factors are also involved in the control of autophagy^19,22,23,37–39^. There is a lot of evidence indicating the reciprocal crosstalk between NF-κB and autophagy, both in physiological and pathological processes. IKK/NF-κΒ signaling axis regulates autophagy in a stimulus- and context-dependent manner and vice versa^6,40,41^. In fact, it has been demonstrated that IKK/NF-κΒ could trigger autophagy by directly inducing the expression of genes or proteins involved in the autophagosome machine, such as Beclin 1, BAG3-HspB8 complex, ATG5, and LC3^40,42,43^. Studies conducted by Comb et al. demonstrated that IKK is also able to induce autophagy, independently of NF-κΒ signaling^44–46^. On the other hand, NF-κΒ can also inhibit autophagocytosis by either increasing the expression of autophagy repressors, like A20, Bcl-2 family members, phosphatase and tensin homolog/mammalian target of rapamycin (PTEN/mTOR) and nitric oxide (NO), or suppressing autophagy inducers, such as BCL-2 interacting protein 3 (BNIP3), JNK1, p53 and ROS^40,47–49^. In turn, autophagy has been shown to regulate NF-κB pathway, mainly by degrading IKK components and NF-κΒ-inducing kinase (NIK) (Fig. 1)^50–54^.

## Autophagy and NF-κB in cancer

In response to different cellular stresses, autophagy plays a pivotal cytoprotective role in maintaining metabolic and cellular homeostasis. Indeed, autophagy substrates are recognized, isolated and degraded in order to meet the energy demand and avoid the accumulation of damaged proteins and organelles^33,34,55^. Paradoxically, recent evidence indicates that autophagy can be also considered itself a mechanism of cell death, hence the term “autophagic cell death”^56,57^. Several in vitro and in vivo studies clarified that this dual biological outcome of autophagy is tissue-specific and it is tightly dependent on the nature of the autophagy substrates^58,59^.

This context-dependent function has been also reported in cancer, where autophagy shows both tumor suppressor and tumor promoter activities^34,60^. In the early phases of cancer development, autophagy has been reported to have a tumor suppressive role, mainly through the blockade of ROS-induced damage and the preservation of cellular homeostasis. Additional mechanisms by which autophagy suppresses carcinogenesis include the preservation of genetic/genomic stability, the maintenance of a normal stem cell compartment, the elicitation of anticancer immune response, and the restraint of inflammation. On the other hand, during tumor promotion, progression and metastasis, autophagy exerts a pro-tumoral activity by both dampening cytotoxic ROS-induced metabolic stress and providing nutrients required for cancer cell survival. In addition, autophagy exerts its tumor-supporting function by increasing the resistance of cancer cells to stressful conditions (i.e. loss of attachment, hypoxia and nutrient deprivation) and therapy-induced dormancy or senescence^33^.

### NF-κB regulates autophagy in cancer cells

The role of NF-κΒ and autophagy in cancer has been well investigated and the impairment of this crosstalk establishes the fate of the cell.

Djavaheri-Mergny et al. reported that in response to TNF-α, NF-κΒ activation represses autophagy in different cancer cell lines including Edwing sarcoma, breast and promyelocytic leukemia^49,61^ via activation of mTOR pathway, a major negative regulator of autophagy. Accordingly, TNF-α treatment induced an increased accumulation of autophagic vacuoles and LC3-II, the conjugated form of LC-3, only in cells lacking NF-κΒ activity. Unsurprisingly, the TNF-α-dependent enhanced autophagy observed in absence of NF-κB occurred via ROS accumulation and a rapid increase in Beclin1 expression^49,62^. Several other studies pointed out the role of NF-κΒ in suppressing the autophagic machinery upon TNF-α treatment, via inhibition of PTEN-mediated suppression of insulin/Akt pathways, which in turn is a potent activator of mTOR pathway^63^.

Recent evidence highlighted the pro-tumorigenic role of NF-κB-induced autophagy in most human cancer. Zhang et al. demonstrated that under stress conditions, transglutaminase (TG2)/NF-κΒ-mediated interleukin-6/signal transducer and activator of transcription 3 (IL-6/STAT3) signaling promotes increased autophagy in mantle cell lymphoma (MCL), thus supporting cell survival. The enhanced macroautophagy in turn, positively regulates TG2/NF-κΒ/IL-6 signaling, thereby implying a potential positive feedback loop required for cancer cell survival. Since increased TG2 levels are associated with poor prognosis in MCL patients, breaking TG2/NF-κΒ/IL-6/autophagy might be a potential therapeutic target for MCL^64^.

In recent years, signalphagy, defined as the termination of cytosolic signaling events by selective autophagy, is catching on in the context of tumorigenesis^65^. Newman and collaborators highlighted that autophagy contributes to carcinogenesis in Ras-mutant cancer cells in vivo through reprogramming gene expression^65^. Selective autophagy of tumor necrosis factor receptor-associated factor 3 (TRAF3) via the cargo receptor nuclear dot protein (NDP52) protein drives nuclear translocation of RelB. Once in the nucleus, RelB represses SMAD-mediated transcription of antitumoral genes downstream transforming growth factor beta (TGF-β) signaling. Accordingly, autocrine and/or paracrine sources of TGF-β dampen tumorigenesis when autophagy is loss in vivo, and this antitumor effect is abrogated by SMAD knockdown^65^. Despite this area is largely unexplored, increased understanding of the mechanisms are needed for targeting signalphagy in cancer.

It is known that in some circumstances IKK complex promotes autophagy, with or without any involvement of NF-κΒ, by inducing the expression of autophagy-regulated genes (i.e*.*, *Atg5, BECN1, LC3*), activating 5' adenosine monophosphate activated protein kinase (AMPK), and/or inhibiting mTOR and p53.

Comb and colleagues demonstrated that IKK activity is required for initiation of autophagy in response to nutrient deprivation, identifying p85α, a regulator subunit of phosphoinositide 3-kinases (PI3K), as mediator of IKK function, both in vitro and in vivo. p85α phosphorylation by IKK results in decreased affinity for tyrosine phosphorylated proteins and PI3K membrane localization, leading to Akt and mTOR inhibition^6,44,45^. Moreover, they also demonstrated that IKK induces autophagic genes, such as *Beclin1*, *Atg5* and *LC3*, and its ability does not require NF-κΒ^45^. In contrast with these findings, it has been demonstrated that IKKα plays an important role in mediating mTOR kinase activation in Akt-active, PTEN-null prostate cancer cells, thus suppressing autophagy^66,67^.

While it is true that NF-κB can regulate the autophagic process, it is also true that autophagy can modulate the NF-κB signaling.

A recent study reported a key role of Beclin1-mediated autophagy in maintaining NF-κΒ and STAT3 constitutively active in human T cell leukemia virus type 1 (HTLV-1)-mediated tumorigenesis. Knockdown of *Beclin1* and selective inhibition of IKK in HTLV-1-transformed T cells reduced growth, due to diminished Tax-induced activities of both transcription factors^68^. In accordance with these roles of autophagy in regulating NF-κB in cancer, Yoon and colleagues demonstrated that enhanced autophagic process and NF-κΒ and STAT3 activation in starved cancer cells caused an increased production of various cytokines and factors including IL-6, interleukin 9 (IL-9), ADAM metallopeptidase domain 12 (ADAM12) and FRAS1 related extracellular matrix 1 (FREM1). In turn, the secretion of these factors led to proliferation, survival and metastasis of cancer cells and migration of endothelial cells contributing to the inflammatory microenvironment surrounding the tumor. Notably, genetic inhibition of autophagic machinery, via *Beclin-1* and *Atg5* silencing, abrogated IL-6 expression, STAT3 phosphorylation, and NF-κΒ activation, suggesting that in this model, NF-κΒ is affected by autophagy^69^.

**Fig. 2:**
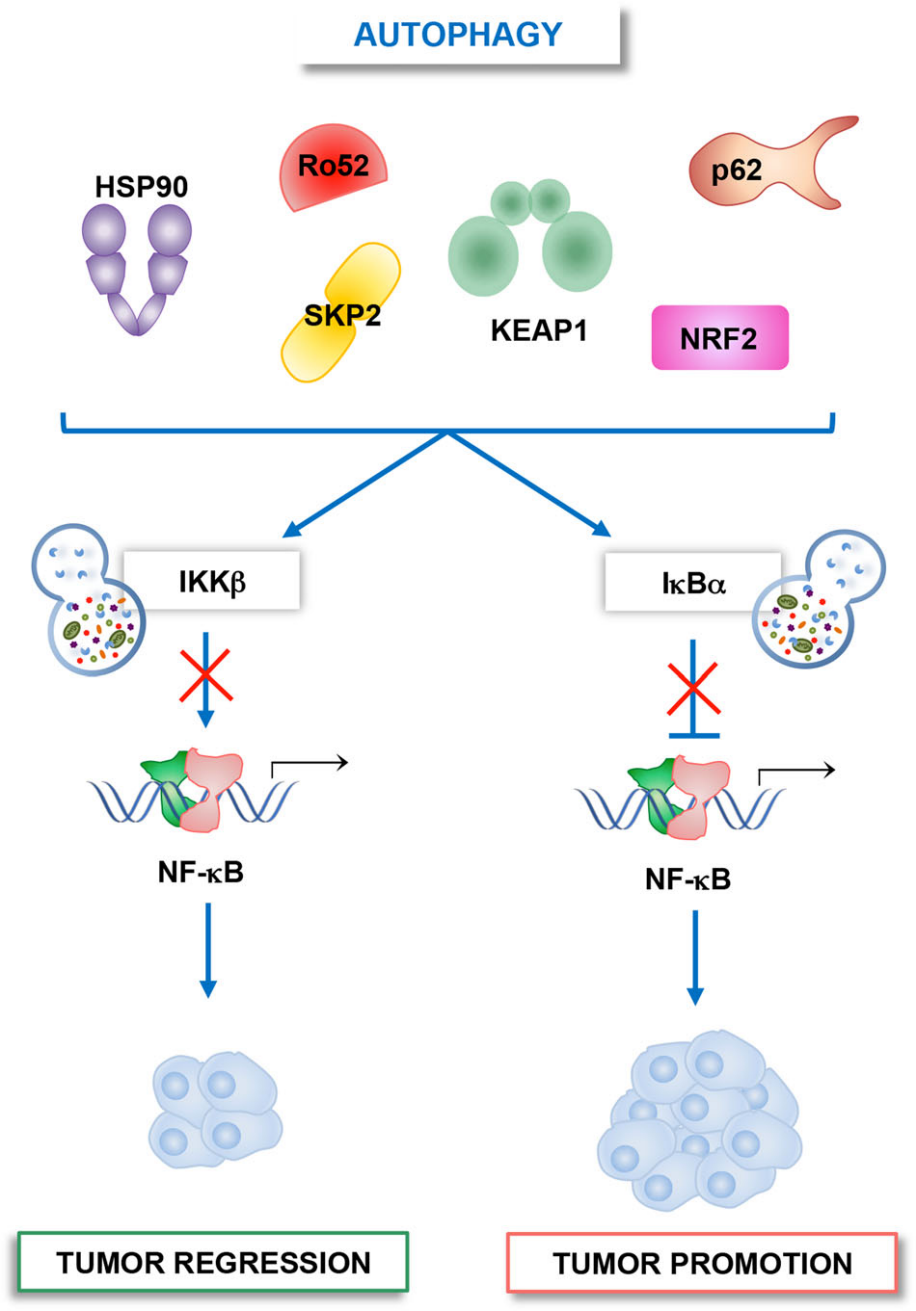
Autophagy regulates IKK/NF-κΒ signaling in cancer cells. Autophagy modulates NF-κΒ pathway by mediating the degradation of either IKKs or IκBα. If IKK is degraded, pro-tumoral NF-κB signaling is inhibited, while the autophagic degradation of IκBα causes NF-κB activation and tumor promotion. The autophagic targeting of core elements of NF-κB pathway involves specific cargo receptors, such as KEAP1, Ro52, Hsp90, SKP2, p62, and NRF2.

It has been reported that autophagy can impact NF-κB activity by mediating either IKKs or IκBα autophagocytosis, thus inhibiting or activating the NF-κB signaling^6,45,46^. The autophagic targeting of NF-κB core pathway components occurs via specific partners, such as Kelch-like ECH-associated protein 1 (KEAP1), E3 ubiquitin ligase Ro52, heat shock protein 90 (Hsp90), S-phase kinase-associated protein 2 (SKP2) and sequestome-1 (SQSTM1/p62) (Fig. 2)^41,70^.

KEAP1 is a ubiquitin ligase, which interacts with nuclear factor erythroid 2-related factor 2 (Nrf2), a transcription factor involved in the inflammatory and antioxidant response. KEAP1 fosters IKKβ ubiquitination and autophagic degradation, thus allowing the downregulation of NF-κΒ signaling^53,71^. Accordingly, with the cytoprotective role of Nrf2 in tumor cells^72,73^, Lee and colleagues demonstrated that proteasome inhibitors favored IκBα degradation by triggering Nrf2-mediated autophagy. Interestingly, proteasome inhibitors induced the upregulation of Nrf2 by both inducing its de novo synthesis and KEAP1 degradation. These events promote NF-κΒ-mediated upregulation of antiapoptotic genes leading to the suppression of apoptotic cell death in lung cancer cells^74^.

Recently Liu and colleagues demonstrated that SKP2, an lipopolysaccharides (LPS)-inducible gene, is able to inhibit NF-κΒ through the degradation of active IKKβ via autophagy. The complex IKKβ−SKP2 is recognized and recruited by the cargo receptor p62. Since SKP2 can be induced by NF-κΒ, this finding suggests that autophagy-mediated SKP2-IKKβ-NF-κΒ axis is modulated through a negative feedback loop^70^. Further evidence demonstrated that autophagy can degrade IKK complex, in particular IKKβ, also following Ro52-mediated mono-ubiquitination^52^.

Another important partner involved in the control of IKK/NF-κΒ axis is Hsp90, a well-known pro-survival cytosolic chaperone involved in the activation of IKK and the switch between autophagy and apoptosis. The study conducted by Qing and colleagues reported that treatment with geldanamycin, an inhibitor of Hsp90, promoted both IKK and NIK degradation via autophagy, resulting in the suppression of NF-κΒ-driven transcription in most cancer cells^50,51,75^. However, under stress conditions, Hsp90 hyperactivation stabilizes its partners and protects cells from cell death^76^.

Moreover, there is some evidence on the role of the autophagy cargo receptor SQSTM1/p62 in modulating NF-κΒ in cancer^77,78^. p62 is a multifunctional protein that exerts its function depending on stimulus and context. Recently, p62 has been identified as a key component required for cancer development and progression, both in vitro and in vivo, and accumulation of p62 is a common event in most human cancer. Accordingly, p62 knockdown reduced both the proliferation of cancer cells and tumor growth. Although the regulation of mitochondrial integrity by mitophagy seems to be the principal mechanism by which p62 promotes cancer cell survival, it remains to be clarified the impact of p62 in the regulation of important signaling pathway in cancer, such as NF-κΒ^60,77,79–81^. p62 alters IKK/NF-κΒ signaling by promoting IκΒα phosphorylation and its autophagy-dependent degradation^20,82,83^. In several human tumors and cancer cell lines increased Ras-mediated p62 overexpression is essential for cell survival and tumorigenesis^78^. In agreement with this, Duran et al. showed that the pro-survival NF-κΒ activation in mouse model of Ras-induced lung adenocarcinoma was dependent on p62. Genetic deletion of p62 impaired NF-κΒ activation, at the level of IKK, and abrogated Ras-driven tumorigenesis, due to increased ROS production and cell death. In addition, the author demonstrated that the lack of p62 enhanced the activation of stress-activated MAPKs, like JNK, and reduced the expression of NF-κΒ-dependent genes, such as ROS scavenger FHC, identifying p62 as a key target in Ras-mediated transformation^77^. p62/NF-κB axis plays also a key role in tumor cell resistance to anticancer drugs. Recently, Yang et al. demonstrated that chloroquine (CQ), an autophagic blocker, triggers the activation of NF-κΒ and its target genes, such as hypoxia-inducible factor 1-alpha (HIF-1α), interleukin-8 (IL-8) and antiapoptotic genes like BCL-2 and BCL-XL, in both squamous cell carcinoma and melanoma cells. Additionally, the authors showed that CQ-induced NF-κΒ activation required autophagosome activation, upregulation of p62 and JNK activation. Knockdown of either p62 or JNK prevented CQ-induced IKK phosphorylation, p62 expression, and NF-κΒ activation. Notably, CQ creates a positive feed-forward loop between p62 and NF-κΒ, the CQ-induced upregulation of p62 actives NF-κΒ, which, in turn, induces p62 expression. Nevertheless, blocking either p62 or NF-κΒ pathway sensitized cancer cells to CQ-mediated apoptotic cell death, suggesting the important role of p62/NF-κΒ axis in mediating cell survival and resistance to CQ in cancer^84^. In keeping with the role of autophagy in drug resistance, Jia and collaborators reported that Bortezomib, a novel anticancer agent used for the treatment of MM, induced autophagy of IκBα in DLBCL, thus promoting cell drug resistance. In this study the authors showed that proteasome inhibitor bortezomib induced canonical NF-κB activation by favouring the accumulation of ubiquitinated proteins, like IκBα, which are subsequently recruited to autophagosomes by p62 carrier. Increased autophagy and ER stress were observed, as demonstrated by elevated LC3-II accumulation, a hallmark of autophagy activation and CHOP, an indicator of ER stress respectively. Treatment with autophagic blocker, CQ, prevented IκBα degradation and bortezomib-induced pro-survival NF-κB signaling, leading to cancer cell death (Fig. 2). Based on these results, a potential way to overcome drug resistance and induce apoptosis of DLBCL cancer cells may be blocking both autophagy and NF-κB pathways^85^.

### NF-κΒ, autophagy, and TME

**Fig. 3:**
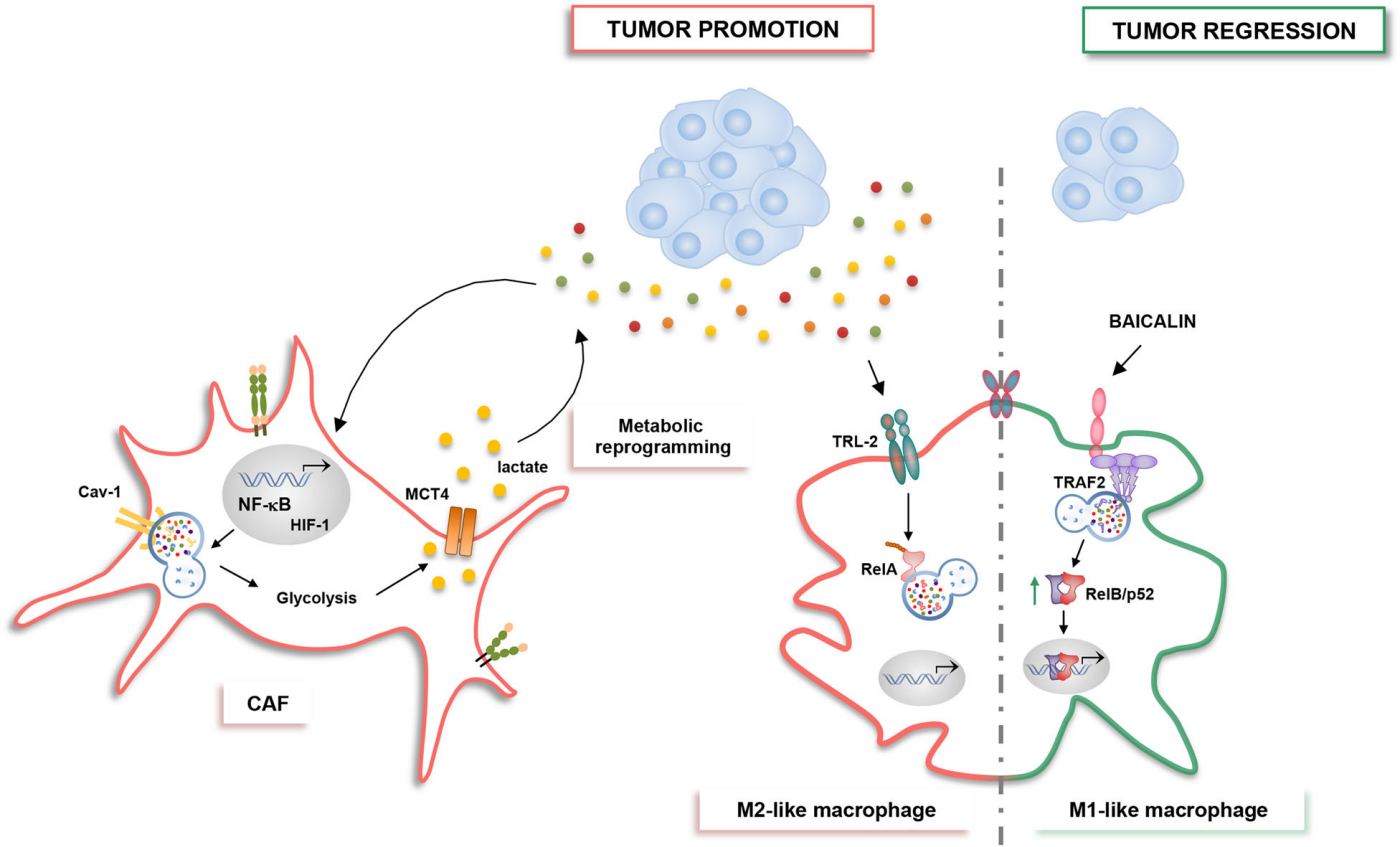
NF-κΒ and autophagy crosstalk in the TME-associated cells can either suppress or promote tumorigenesis. Reactive oxygen species (ROS) generated by cancer cells promotes NF-κΒ and HIF-1 activation in CAFs. In turn, these two transcription factors induce the autophagic degradation of caveolin 1 (Cav-1). Consequently, loss of Cav-1 amplifies oxidative stress and triggers glycolytic catabolism and lactate extrusion, thus promoting the anabolic growth of adjacent cancer cells. In TAMs, the autophagic degradation of RELA/p65 induced by TLR2 activation promotes the M2-like phenotype in the context of HCC. On the contrary, the lysosomal degradation of TRAF2 induced by baicalin treatment determines the transcriptional activation of RelB/p52 pathway, thus reprogramming TAMs towards the pro-inflammatory M1-like phenotype and counteracting HCC.

The complex role of the tumor-microenvironment (TME) in promoting cancer cell survival and immune evasion has been characterized as a new hallmark of cancer^86^. The dynamic crosstalk between TME and tumor cells affects cell survival and tumor progression, but it remains hard to fully understand how the autophagy fosters the cross talk between tumor and inflammatory cells. It is known that NF-κΒ is an important player during inflammatory response and tumorigenesis as well as in the polarization of tumor-associated macrophages (TAMs)^23,87–91^. Recently, has been reported that autophagy has both anti- and pro-inflammatory effects in the TME by modulating NF-κΒ pathway^92^. Chang et al. demonstrated that Toll-like receptor 2 (TLR2) promoted the M2-like phenotype of hepatoma-derived macrophages. In addition, they showed that NF-κΒ activation was inhibited by TLR2-dependent selective autophagic degradation of NF-κΒ/p65-containing aggresome-like structures (ALS), through the autophagy cargo receptor, SQSTM1/p62. This event occurred in hepatoma-derived M2-macrophages, but not in M1-polarized macrophages, both in vivo and in vitro. The inhibition of autophagy by lysosomal inhibitor bafilomycin A1 or knockdown of *Atg5* restored NF-κΒ activity and induced pro-inflammatory cytokines, switching macrophages from M2 to M1 phenotype. These data suggest that autophagy is a player in “re-educating” macrophages and regulating macrophage-associated antitumor immune response^93,94^. Recently, it has been demonstrated that after treatment with Baicalin, a natural flavonoid, autophagy-induced RelB/p52 activation, a constituent of the alternative NF-κΒ pathway, induced TAMs repolarization towards M1-like phenotype, resulting in the suppression of HCC in vivo. Importantly, Tan et al. showed that Baicalin acted specifically on M2 macrophages, while no affecting M1 phenotype in the TME. They also observed that downregulation of TRAF2 via lysosomal degradation regulated macrophage polarization towards pro-inflammatory phenotype and increased transcriptional activation of RelB/p52 pathway. Inhibition of both autophagy and RelB rescued the effects of baicalin, suggesting that this natural compound might be a potential immune therapeutic candidate for cancer therapy, with particular regard to HCC (Fig. 3)^95^.

Recently, Sun et al. demonstrated that *Atg5* loss in Kupffer cells, the liver resident macrophages, at early stages of tumorigenesis speeded up fibrosis, inflammation and finally HCC. This pro-tumoral effect associated with autophagy impairment was due to increased ROS production and NF-κB/interleukin-1 alpha/beta (IL-1α/β) signaling, resulting in inflamed TME, thus demonstrating an antitumorigenic role of Kupffer cell-associated autophagy in HCC tumor initiation^96^.

In accordance with the relevance of TME-associated autophagy in cancer, Martinez-Outschoorn et al. showed that, under oxidative stress and hypoxia, cancer cells induced autophagic degradation of caveolin 1 (Cav-1) in cancer-associated fibroblasts (CAFs) by upregulating NF-κB and HIF-1. Importantly, the authors demonstrated that cancer cells promoted NF-κΒ and HIF-1 activation in adjacent fibroblasts in a paracrine manner, and that the pharmacological inactivation of both HIF-1 and NF-κΒ prevented Cav-1 degradation. In addition, as an important structural protein and nitric oxide synthase (NOS) inhibitor, loss of Cav-1 in the stroma triggered glycolytic catabolism and lactate extrusion, via HIF-1 stabilization and upregulation of monocarboxylate transporter 4 (MCT4). This catabolic phenotype of stroma cells, in turn, promotes anabolic growth of adjacent cancer cells, thus highlighting the importance of this metabolic synergy in driving cancer (Fig. 3)^97,98^.

Although further investigations are needed to best characterize the roles of NF-κΒ and autophagy during tumorigenesis, its involvement in both cancer cells and TME might have profound implications to oncogenesis and anticancer therapy.

## Necroptosis and NF-κB

Cells react to cellular stress, pathogen infection, and organismal development undergoing distinct forms of cell death^99^. Research on the mechanisms of PCD induced following the interaction of a death ligand with its death receptors (DRs) expressed on the cell surface has revealed a signaling pathway that triggers programmed necrotic cell death or necroptosis. These receptors, including tumor necrosis factor receptor 1 (TNFR1), Fas (CD95/APO-1) and TNF-related apoptosis-inducing ligand (TRAIL), show a cytoplasmic death domain (DD) through which a regulated death signal could be transduced, resulting in apoptotic or necroptotic cell death^100,101^. Although the necroptosis outcome is phenotypically similar to necrosis, as the final event leads to a lytic cell death, it is defined by a dedicated signaling cascade. Unlike extrinsic apoptosis, which causes caspase activation, death via canonical necroptosis is induced by the activation of the receptor-interacting protein kinases RIPK1-RIPK3 and the pseudokinase mixed lineage kinase domain-like (MLKL).

Classically, under apoptosis-deficient conditions, e.g. inactivation of Caspase-8 activity, or pharmacological inhibitors, the activated RIPK1 interacts with RIPK3 through RHIM domains forming the heteroamyloid complex called necrosome^102–104^. This platform is considered to be important, as it can sustains and amplifies key signals that otherwise might be decreased by protein degradation. The RIPK1-RIPK3 oligomerization leads to RIPK3 auto-activation that, in turn, phosphorylates MLKL. MLKL phosphorylation is the important execution step of necroptosis downstream of DR ligation and it is required for its translocation to the membrane, where it generates cation channels causing plasma membrane rupture, induces phosphatidylserine (PS) externalization and release of pro-inflammatory intracellular components^105–107^. The release of the cellular content into the extracellular space exposes several molecules that function as damage-associated molecular patterns (DAMPs) to the immune system through pattern recognition receptors (PRRs), thus promoting, perpetuating, and potentiating the inflammatory response. Such DAMPs include lactate dehydrogenase (LDH), high-mobility group box 1 (HMGB1), mitochondrial DNA, N-formyl peptides, interleukin-1 (IL-1) and interleukin-33 (IL-33)^108,109^.

The current model is that necroptosis can function as an alternative cell death pathway which eliminates caspase-deficient cells in the event of infection, while, at same time, releases endogenous molecules and newly inflammatory signals generated during cell death to recruit and activate immune cells at site of necroptotic cell^110,111^.

As previously discussed, NF-κB pathway plays crucial role in regulating both inflammatory processes and programmed cell death, and, unsurprisingly, it is also involved in necroptosis.

Yatim et al. formally demonstrated for the first time that the release of endogenous DAMPs (ATP and HMGB1) induced by RIPK3/MLKL-mediated cell lysis was not sufficient for CD8^+^ T cell priming, but the transcriptional NF-κB activation in the dying cell was necessary to achieve T cell priming. The NF-κΒ regulates the expression of many pro-inflammatory genes that may actively contribute to immunogenicity of dying cells. Although the mechanism whereby NF-κB activation within dying cells actively regulates cross prime and the molecular signals involved in this process remain to be elucidated, it has become apparent that this regulation contributes to the immunogenicity of necroptotic cells^108^.

Moreover, NF-κB signaling can be triggered by RIPK1, which acts as molecular switch that can induce inflammation and cell survival, as well as apoptosis or necroptosis, depending on the context. Indeed, as a scaffold molecule, RIPK1 mediates the activation of NF-κB downstream of DR ligation by recruiting key activators of NF-κB pathway, such as the TGF-β-activated kinase 1 binding protein 1/2 (TAB1/2) and NEMO, to promote the activation of TAK1 and IKK complex^112,113^. The consequent NF-κB-mediated expression of pro-survival genes, such as cFLIP, prevents extrinsic apoptosis by inducing the formation of inhibitory cFLIP/caspase-8 heterodimers, thus inhibiting caspase-8 activation^114,115^. In the absence of NF-κB, active dimers of caspase-8 are formed driving extrinsic apoptosis^116,117^. Active caspase-8 not only initiates the apoptotic program, but also negatively regulates necroptosis by cleaving and inactivating essential necroptosis mediators such as RIPK1 and RIPK3^117^. However, depending on the context, RIPK1 can also promote the activation of caspase-8. Complex post-translational modifications tightly regulate the integration of RIPK1 into distinct multiprotein signaling complexes that will ultimately decide the cell fate^118–120^. Such modifications include ubiquitination and phosphorylation but also caspase-mediated cleavage. However, the possibility that RIPK1 shifts from pro-survival to pro-apoptotic and -necroptotic signaling depends on the intracellular RIPK3 levels. Since RIPK3 expression is cell-type restricted and a high intracellular concentration of this factor is required for initiating the necroptotic program, RIPK3 represents a key determinant to transduce necroptosis signal through the canonical RIPK1-RIPK3-MLKL axis (Fig. 1)^103,121^.

## NF-κB and necroptosis in cancer

Evading cell death is considered one of the characteristics of cancer cell. The apoptotic evasion or downregulation of the apoptotic regulators contributes to the disease progression of many cancers^122^. Instead, there is less knowledge regarding the role of endogenous necroptotic signaling within the tumor cell, mostly because the study of necroptosis has been complicated by the absence of specific markers that can be used in vivo^123^. Transient events such as phosphorylation of RIPK3 and MLKL are not easily detectable in the context of human or murine tumor models.

The TNF-α-induced pro-survival function of NF-κΒ in cancer cells is recognized as a main feature in most human cancers^27,124–126^. Defective activation of NF-κΒ (i.e*.*, genetic deletion or pharmacological inactivation) can promote apoptosis or, under caspase-8-deficiency, necroptosis^99,127–129^. In this context, Hernandez et al. demonstrated that blocking NF-κB in OVCAR3 cells, one of the few ovarian cancer cell lines expressing RIPK3, resulted in TNF-α-dependent cell death. While cancer cells proficient for caspase-8 died by apoptosis, the depletion of caspase 8 promoted necroptosis by stabilizing RIPK1. Of note, although the authors proposed a potential role of necroptosis to overcome resistance to apoptosis and improve survival of those patients exhibiting low caspase-8 expression, it is now clear that a limit could be the absence of functional necroptotic machinery too^130^.

The role of necroptosis in mediating cell death has been well characterized in many human diseases^131^, although is not well defined the interaction between IKK/NF-κΒ and RIPK1 in apoptosis and necroptosis. Several data prompt at the existence of organ-specific ways in cell-death inducing stimuli that could be dependent from the levels of expression of the proteins mainly implicated in cell survival or cell death induction.

**Fig. 4:**
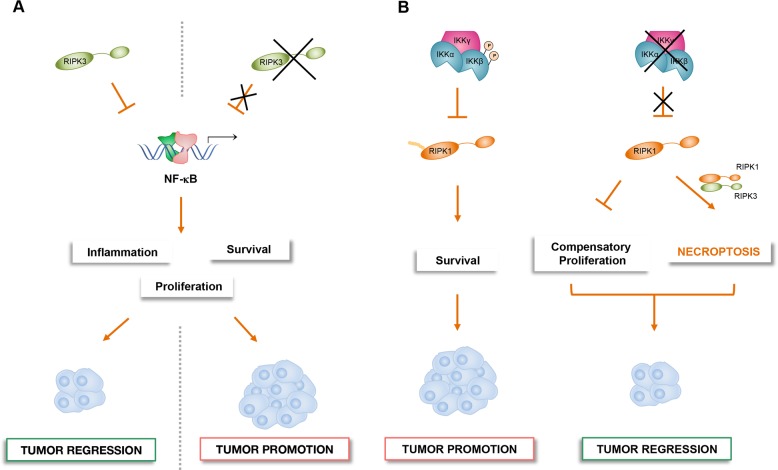
The role of NF-κΒ and RIPK1/RIPK3-mediated signaling in tumorigenesis. **a** RIPK3 activation in intestinal epithelial cells induces necroptosis, thus counteracting CRC development. On the contrary, RIPK3 loss in the same cells leads to excessive activation of several pathways including NF-κΒ, which result in abnormal proliferation and occurrence and development of CRC. **b** In the liver, activated IKKα and IKKβ directly phosphorylates RIPK1 and inhibits RIPK1-dependent PCD, thus promoting hepatocarcinogenesis. By contrast loss of IKKα/β-dependent RIPK1 phosphorylation prevents carcinogenesis by suppressing compensatory proliferation of hepatocytes and increasing necroptosis cell death.

Recently, Bozec and colleagues demonstrated that necroptosis, in particular RIPK3, suppressed inflammation-driven CRC^132^. Accordingly, tumor cells lacking RIPK3 were more invasive, both in vitro and in vivo. The authors showed that RIPK3 loss induced tumorigenesis via upregulation of NF-κΒ, STAT3, AKT, and Wnt-β-catenin pathways activation, thus in turn leading to aberrant tumor cell proliferation, colon inflammation, immune cell infiltration and finally CRC. The upregulation of NF-κΒ in RIPK3-deficient mice induced the expression of cytokines (i.e*.*, IL-6, TNF-α, IL-1β) and chemokines (i.e*.*, CCL2, CXCL1, CXCL2), as well as the transcription of genes involved in survival, cell cycle progression. This pro-tumorigenic milieu fosters the transition from inflammation to CRC malignancy. The capability of RIPK3 to negatively regulate the activation of different signaling pathways, such as NF-κΒ, together with its antitumoral function identified RIPK3 as a tumor suppressor in CRC. Accordingly, reduced expression levels of RIPK3 observed in CRC patients support the idea that RIPK3 plays a pivotal role in CRC pathogenesis but further studies are needed to clarify the tumor suppressor function of RIPK3-dependent necroptosis in CRC tumorigenesis (Fig. 4a)^132^.

In the liver, the IKK/NF-κB signaling pathway has emerged as a principal regulator of homeostasis and disease. Complete deletion of NF-κB-mediated transcription in liver parenchymal cells (LPCs) in mice (*RelA*^*LPC*-^^*KO*^ or *RelA/RelB/c-Rel*^*LPC*-*KO*^ mice) did not cause spontaneous liver damage^133,134^, in contrast NEMO deletion induced liver damage with massive compensatory proliferation and spontaneous development of HCC^135^.

In human, NEMO expression is completely absent in 40% of HCC biopsies studied (85 samples) when compared with adjacent normal tissue. Indeed, the correlation between the loss or low protein expression of NEMO and poor prognosis for patients has been reported^136^, emphasizing the idea that NEMO-dependent survival functions may be critical for the prevention of chronic liver damage and HCC in at least a certain group of patients.

The expression of a kinase inactive mutant of RIPK, in a contest of *NEMO*^*LPC*^ deletion, prevented cell death and HCC in mice, this evidence strongly suggested the interplay between NEMO and RIPK1 kinase-activity-dependent apoptosis in liver disease, whereas the role of the necroptosis remains controversial^133^.

In according with the antitumoral function of necroptosis, Koppe and colleagues demonstrated that IKKα and IKKβ deletion in LPC (*IKKα/β*^*LPC-KO*^) induced cholestasis and, at the same time, inhibited HCC, due to increased intrahepatic necrosis and reduced compensatory proliferation in vivo. They also showed that both RIPK1 and RIPK3 promoted necroptosis in *IKKα/β*^*LPC-KO*^ mice, as demonstrated by reduced necrotic foci in livers after additional deletion of *Ripk3* and *Ripk1*, suggesting that these two kinases play a role in mediating necroptosis in these mice. Furthermore, Koppe et al. demonstrated that RIPK1 is the main players of the NF-κΒ-independent *IKKα/β*^*LPC-KO*^ phenotype of mice characterized by cholestasis, reduced compensatory proliferation and then carcinogenesis, underlying a role of the catalytic IKKs in controlling RIPK1 activity independently of NF-κΒ (Fig. 4b)^137^. In a recent study, Koppe et al. showed that the additional deletion of *NEMO* in the *IKKα/β*^*LPC-KO*^ mice (*IKKα/β/NEMO*^*LPC-KO*^) prevented necroptosis of LPCs and supported apoptosis and compensatory proliferation of both hepatocytes and cholangiocytes thus inducing HCC^138^. This study would support the idea that NEMO protein alone can directly inhibit the activation of apoptosis mediated by RIP1 Kinase activity in a context of NF-κB inhibition.

Although previous studies demonstrated that NEMO induces necroptosis by promoting RIPK1/RIPK3 necrosome^139,140^, further studies are needed to better understand the IKK/NF-κΒ-independent role of NEMO in controlling PCD in the liver. Nevertheless, based on these studies, the use of small molecule inhibitors of RIPK1 Kinase, also blocking necroptosis, would be an effective therapeutic option for patients with liver damages and HCC.

In the last few years, the immunogenicity of necroptotic cancer cells and its possible role in antitumor immune responses has attracted enormous attention as an alternative strategy for eliminating cancerous cells. As previously said, necroptosis induces adaptive immune responses by releasing DAMPs in the TME which in turn stimulate DCs and macrophages to secrete pro-inflammatory cytokine important to activate cytotoxic CD8^+^ T cells as well as interferon-gamma (INF-γ) in response to tumor antigen stimulation^108,141–143^. One of the specific characteristics of a cell undergoing necroptosis is that, apart DAMPs release, it can synthesize proteins and secrete antigens and pro-inflammatory cytokines, such as IL-6. Hence, the necroptotic environment becomes enriched also in pro-inflammatory cytokines/chemokines which might lead to immunostimulation and immunogenicity of necroptotic cancer cells. In keeping with these findings, Schmidt et al. showed that RIPK3-dependent-necroptotic cervical cancer cells induced the expression of IL-1α which stimulate the activation of DCs. As a result, activated DCs, in turn, promote the upregulation of DC surface markers as well as the release of interleukin-12 (IL-12) leading to antitumor effects^144^.

**Fig. 5:**
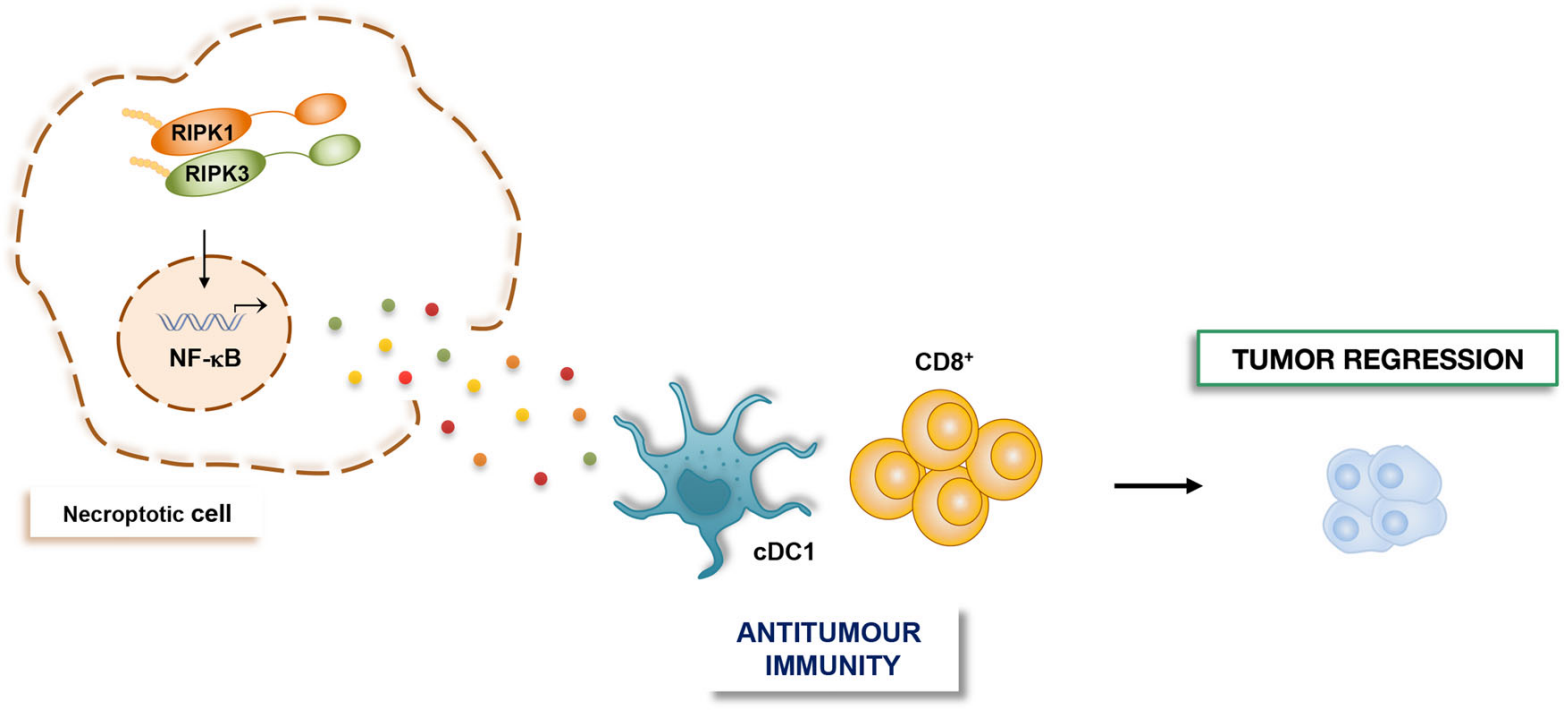
NF-κΒ and necroptosis control antitumor immune response within the TME. RIPK1/RIPK3 necrosome complex induces NF-κΒ-dependent cytokines production in necroptotic cells. These NF-κΒ-dependent paracrine signals, in turn, promote antitumor immunity by activating cDC1 and CD8^+^ cells within the TME.

Recently, the role of RIPK1 and RIPK3 within the TME has been described by Snyder et al. The authors demonstrated that ectopic injection of necroptotic cells into tumors induced RIPK1/RIPK3-mediated production of NF-κΒ-dependent cytokines. These factors, together with conventional dendritic cells 1 (cDC1s) and CD8^+^ T cells, promoted in situ and systemic antitumor immune response and tumor regression. Importantly, inhibition of NF-κΒ activation via BAY-117085, significantly reduced both the tumor control effects and the survival advantage, demonstrating that intact NF-κΒ transcriptional signaling downstream of RIPK1/RIPK3 activation is required for the immunogenicity of necroptotic cells in the TME (Fig. 5). Furthermore, immune-mediated tumor control occurred independently of MLKL, cell lysis, and DAMPs release. They also suggest that necroptosis in the TME can synergize with α-prohrammed cell death protein 1(PD-1) to promote durable tumor clearance in vivo^145^.

Taken together, these studies indicate that necroptosis is immunogenic, extending the current concept of immunogenic cell death, and open ways for the development of new cancer therapy strategies.

## Conclusion and perspectives

Apoptosis, autophagy, and necroptosis are interconnected processes, which intersect with NF-κB signaling in response to different stimuli. This interplay exists also in cancer, thus opening the door to new therapeutic opportunities. It has been demonstrated that autophagy modulates the sensitivity to anticancer drugs^146^ and is able to switch its function upon the context^147^. Over the last few years several studies showed that combination of inhibitors of autophagy and survival pathways as NF-κB could be an important therapeutic option to sensitize tumor cells to apoptotic cell death^148–150^, thus improving cancer patient survival. Although the role of necroptosis in cancer is controversial, the fact that many tumors cells lack key necroptotic components^132,151–154^ suggests that restoring necroptosis might provide an intriguing opportunity for developing new anticancer treatments to bypass apoptosis resistance and promote strong antitumoral response in cancer therapy.
